# Behavioural interventions for people living with adult-onset primary dystonia: a systematic review

**DOI:** 10.1186/s12883-016-0562-y

**Published:** 2016-03-22

**Authors:** C. J. Bernstein, D. R. Ellard, G. Davies, E. Hertenstein, N. K. Y. Tang, M. Underwood, H. Sandhu

**Affiliations:** Division of Health Sciences, Warwick Medical School, University of Warwick, Coventry, UK; Warwick Clinical Trials Unit, Warwick Medical School, University of Warwick, Coventry, UK; University of Southampton, Southampton, UK; Department of Psychiatry and Psychotherapy, University of Freiburg Medical Center, Freiburg, Germany; Department of Psychology, University of Warwick, Coventry, UK

**Keywords:** Idiopathic adult onset dystonia, Behavioural interventions, Self-management, Quality of life

## Abstract

**Background:**

Primary dystonia is a chronic neurological movement disorder that causes abnormal muscle movements. Pain and emotional distress may accompany these physical symptoms. Behavioural interventions are used to help people with long term conditions improve their quality of life. Little is known about behavioural interventions applied to Dystonia. We report a systematic review of studies reporting current evidence of behavioural interventions for people with primary dystonia.

**Methods:**

We did systematic searches of Medline, PsycINFO, AHMED and CINAHL. We assessed the methodological quality of included studies using a risk of bias tool. Any disagreements were resolved by liaising with an independent rater. Physiological outcomes such as dystonia severity and psychological outcomes such as sleep and depression were selected on the basis that primary dystonia causes motor and non-motor symptoms. No time limit was placed on the searches. A narrative synthesis of the results is presented.

**Results:**

Of 1798 titles and abstracts screened, 14 full articles were retrieved and inclusion and exclusion criteria applied. Of these a final nine were eligible for the review (*N* = 73). Only two were Randomised Controlled Trials (RCTs). Using the Movement Disorders Society (MDS) dystonia classification, that was published after this work started, all of the included studies were of idiopathic adult onset focal dystonia without associated features. These included: blepharospasm (eye dystonia) (*N* = 1), cervical dystonia (neck dystonia) (*N* = 2), writer’s cramp (hand dystonia) (*N* = 3) and the yips (*N* = 3). No studies reported on dystonia that affects two or more body regions. Studies reported good adherence and response rates to treatment. Physiological and psychological improvements were noted in all studies at weekly, monthly and yearly follow-ups. Caution should be taken when interpreting the results because of the scarcity of RCTs identified, use of small sample sizes, and inappropriate statistical methods.

**Conclusion:**

We identified few studies; mainly of poor methodological quality that all studied a focal dystonia. It is not possible to draw firm conclusions. Nevertheless, the data suggests that a combined behavioural therapy approach including relaxation practice for people with idiopathic adult onset focal dystonia merits further investigation.

## Background

Dystonia is a neurological condition that can cause abnormal and repetitive muscle contractions and spasms [1]. It is the third most commonly diagnosed movement disorder after Parkinson’s disease and essential tremor [2] with a prevalence variously estimated to be in range of 30–7320 per million [2]. The wide variations on prevalence estimates reflect the different approaches and definitions used by different authors.

Idiopathic adult onset dystonias affecting one body part such as the hand (writer’s cramp) or eyes (blepharospasm) are known as focal dystonia and usually affect adults [1]. In contrast, generalised dystonia typically occurs during childhood and causes spasms and cramping in two or more affected body regions [1]. There may be genetic associations with some idiopathic adult onset dystonias; however, in a majority of cases the cause of disease is unknown [1].

Treatments for generalised and focal dystonia, including pharmaceutical or surgical options, focus predominately on the motor symptoms of the condition. Conservative therapies that have been suggested for helping people with idiopathic adult onset dystonia include: acupuncture, biofeedback, chiropractic manipulation, osteopathy, physiotherapy, speech therapy and transcutaneous electrical nerve stimulation (TENS) [3–6].

However People living with idiopathic adult onset dystonia can experience low self-efficacy, social withdrawal, a negative self-concept and subsequently, poor health outcomes and quality of life [7–10].

More specifically non-motor symptoms of dystonia can include impact on mood (low mood including depression) and emotions, cognitive functioning (such as memory) and impaired sleep [11] Similarly as with other conditions such as Parkinson’s disease, the non-motor symptoms dominate clinical impact and can lead to severe disability and an impaired quality of life [12].

A systematic review investigating the relationship between mental health and ‘primary and other genetic forms of dystonia’ found a relationship between dystonia and emotional disturbance, dystonia and psychiatric disorders and dystonia and cognitive difficulties [11].

People with cervical dystonia for example have a 91.4 % chance of meeting the criteria for a psychiatric illness compared to 35 % in the general population [13]. Other psychiatric illnesses include; major depression, social phobia, panic disorder and for these with focal dystonia a higher risk of obsessive compulsive disorder and anxiety disorder [14]. There is some evidence that cognitive behavioural therapy is effective in the use of the management of anxious thoughts and mood related to muscle tension which could lead to better patient outcomes, however the evidence is limited [15].

We report a systematic review of the evidence for all behavioural interventions for adults living with idiopathic adult onset dystonia.

## Methods

The focus of this review is adults living with primary dystonia (idiopathic adult onset dystonia). We excluded studies of people with acquired dystonia to ensure we were reporting dystonia specific effects rather than the combined symptoms from any acquired disorder (e.g., psychiatric, infectious, toxic or neoplastic) [1].

When we developed the protocol for this review we used the terms then current and set our parameter for study entry to be a primary dystonia; this was the terminology used in our PRoSEPRO registration. Since then the Movement Disorder Society‘s new classification has been published. We did not exclude any studies on the basis of clinical features; axis one of the Movement Disorder Society (MDS) classification. In axis two of the MDS classification our entry criteria map onto the idiopathic domain which includes both sporadic and familial cases. We excluded studies of people with known nervous system pathology, known degenerate causes, and those with acquired causes from axis 2 of the MDS classification.

We have post-hoc classified the included studies according to the new MDS criteria. We have, however, kept our original terminology elsewhere in the paper.

Nearly all of the studies we identified had substantial methodological limitations including poor study designs, small samples, and inappropriate statistical tests. Therefore, we were unable to conduct a rigorous meta-analysis.

### Search strategy

**Fig. 1:**
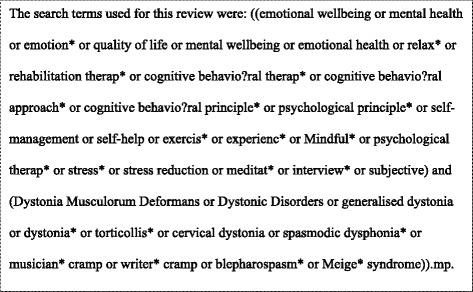
List of review search terms

Between December 2014 and January 2015 we searched: Medline, PsycINFO, AHMED and CINAHL. No date restrictions were applied. Primary dystonia is a broad category encapsulating several different types of medical conditions but studies on dystonia are inconsistently coded in publication databases. For this review, we constructed a broad (sensitive) search strategy to identify relevant studies (please refer to Fig. 1).

### Selection criteria

Relevant outcome measures included in this review are: wellbeing and/or quality of life and/or psychological functioning and/or sleep and/or other clinical measures such as dystonia severity and/or pain. We included studies of adults (≥18) reporting on individuals living with primary dystonia that examined the effect of behavioural interventions on relevant outcomes. Behavioural interventions are defined as interventions where patient behaviour is the key and the main aim to change that behaviour and can be applied at an individual level, community level or international level [16] (and can include highlighting thoughts processes, beliefs and appraisals and learning to challenge and modify these to improve and change behaviour [15].

We included studies published in English, French, German, Italian and Punjabi. Studies were excluded if they conducted research with individuals under the age of 18 years, animals and secondary types of dystonia or dystonia with another primary condition. Studies of non-behavioural - interventions e.g., medical treatments and psychodynamic therapy were excluded.

We included all primary research reporting quantitative outcome; irrespective of study design. Non-primary articles including secondary analyses, review papers, guidelines, statements, meeting summaries or comments were excluded.

Two independent reviewers (DE and CB) screened the studies’ titles and abstracts for eligibility. For potentially eligible studies’, full-texts were then screened by the rest of the team with the exception of MU who acted as an independent adjudicator, making a final decision on any disputed texts.

### Assessing methodological quality

Assessments on all identified studies were performed using a risk of bias rating table that was adapted from a recent systematic review on sleep in primary dystonia [17]. The criteria of risk were based on: diagnosis process, sample size, use of a control group, relevant and valid outcome measures, sufficient description of the intervention, medication and statistics. Risk of bias for each study was rated according to a ‘low’, ‘moderate’, ‘high’ or ‘unclear’ score.

### Data extraction

Socio-demographic and clinical details of study participants were extracted by CB and reviewed by the rest of the team for accuracy. Information about the studies’ research question(s), setting(s), behavioural intervention(s), method(s) and outcome measure(s) were also recorded using a pro-forma. The results of the studies were extracted to evaluate psychological (e.g., quality of life, well-being, sleep) and clinical (e.g., pain, dystonia severity, functioning) outcome measures in relation to the reported behavioural intervention(s). A narrative synthesis of the results is reported.

## Results

**Fig. 2:**
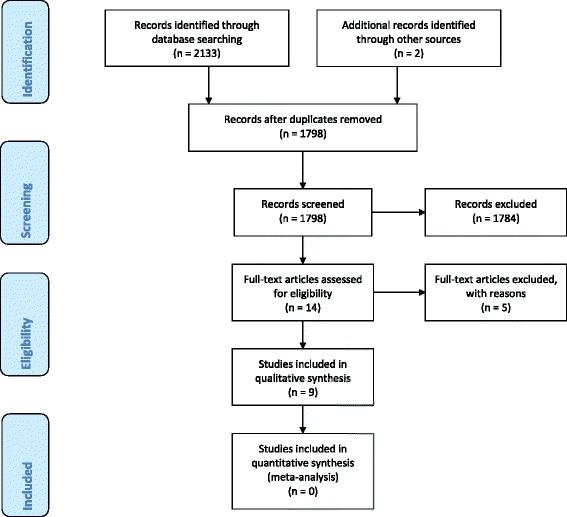
Flow diagram of screening and identification process [20]

The searches retrieved from the databases yielded 2133 hits. Two studies from one included article’s [18] reference list were identified and the abstracts reviewed; one was potentially eligible but the authors were unable to locate the full-text article and so it has been excluded [19]. We examined 14 full papers and included nine: (Fig. 2 [20]).

We found limited evidence of the following behavioural interventions for different idiopathic adult-onset focal dystonia types: Solution-Focused Guided Imagery (SFGI) for the yips [18, 21, 22], exercise and relaxation practice [23] and Cognitive Behavioural Therapy (CBT) [24] for cervical dystonia, relaxation, systematic desensitisation (SD), assertiveness training and/or Electromyography (EMG) feedback [25], habit reversal with in vivo exposure, awareness training and re-education [26] and relaxation or habit reversal therapy [27] for writer’s cramp and conditioning therapy and relaxation practice for blepharospasm [28]. No studies reported on generalised dystonia. Of the nine studies we identified, two were Randomised Controlled Trials (RCTs) [23, 27] utilising small sample sizes. One RCT found a significant finding for improvement in handwriting quality in both the intervention and control groups at follow-up (week 20) [27]. While all studies reported improvements in dystonia severity and/or emotional distress at weekly, monthly and yearly follow-ups, the poor methodological quality of the data means that firm conclusions cannot be drawn about the effectiveness of behavioural therapies for people living with idiopathic adult onset dystonia.

### Quality assessment

Table 1 provides an overview of the findings. Please see web Appendix for the criteria by which the studies were judged (adapted from Hertenstein et al. [17]).

**Table 1:** Quality assessment summary

	1	2	3	4	5	6	7
Bell and Thompson [18]	Moderate	High	High	Moderate	Moderate	Unclear	Low
Bell et al. 21]	Moderate	High	High	Moderate	Moderate	Unclear	High
Bell et al. [22]	Low	High	High	Moderate	Low	Unclear	High
Boyce et al. [23]	High	High	Moderate	Low	Moderate	Moderate	High
Cottraux et al. [25]	High	High	High	High	High	Unclear	Low
Faircloth and Reid [24]	Unclear	High	High	Moderate	Moderate	Unclear	Low
Greenberg [26]	Unclear	High	High	Moderate	Moderate	Unclear	Low
Sharpe [28]	Unclear	High	High	Unclear	High	Moderate	Unclear
Wieck et al. [27]	High	Moderate	Moderate	Moderate	Moderate	Unclear	Moderate

1. Diagnostic process-How well was the diagnostic process described?

2. Sample size-Is this defined?

3. Control group-Is a control group included and if so how well is it defined?

4. Physiological/psychological measure-To what extent are validated outcome measures described/used?

5. Description of intervention-How clear is the description of the intervention?

6. Medication-How well are the medications described?

7. Statistics-How appropriate is the statistical approach?

*Low* low risk of bias; *moderate* moderate risk of bias, *high* high risk of bias, *unclear* unclear risk of bias

Adapted from Hertenstein et al. [17]. See also web Appendix

We found two RCTs (*N* = 20) [23] and (*N* = 23) [27], two are described as using multiple baseline across subjects design (*N* = 4) (which is when the same treatment is staggered across participants and multiple data points are obtained from the baseline, intervention and follow-up phases) [21, 22], and two case studies (*N* = 1) [24, 28]. The remaining three were described as being: a case study using a multiple baseline across situations design (*N* = 1) [18] (which includes studying the same behaviour across different circumstances, with an aim to test the relationship between treatment and behavioural change in a single subject), an uncontrolled multimodal study (drawing on different modalities including relaxation, systematic desensitization and assertiveness training and biofeedback which was tailored to participants), (*N* = 15) [25], and a clinical study (reporting the clinical treatment of four cases, delivered by a trained therapist) (*N* = 4) [26] (Table 2).

**Table 2:** Included studies basic characteristics and sample

Citation	Setting (Country and location)	Design	Sample size (N=)	Mean age (Years)	Gender (m) (f)	Ethnicity	AAO^a^ (Years)	MD^b^ (Years)	PM/T^c^
Bell and Thompson [18]	Golf course	CS	1 (case study)	40y	1 m	Caucasian/“White”	NR	3y	NR
Bell et al. [21]	USA, golf courses	CS	*N* = 3	51y	3 m	Caucasian	NR	NR	NR
Bell et al. [22]	USA, golf courses	CS	*N* = 4	51y	4 m	Caucasian	NR	NR	NR
Boyce et al. [23]	Australia, Physiotherapy out-patients clinic & and at home.	pRCT	*N* = 20 intervention group *N* = 9; control group *N* = 11	57.8y	70 % f; 30 % m	NR	NR	10.2y	Botulinum toxin therapy injections
Cottraux et al. [25]	unspecified,	pRCT	*N* = 15 Multimodal treatment *N* = 9; (EMG) feedback *N* = 4	36y	27 % f, 73 % m	NR	NR	3.6y	psychotherapy
Faircloth and Reid [24]	England, unspecified	CS	1	36y	1 m	NR	26y	10y	Botulinum Toxin therapy injections
Greenberg [26]	England, London, participants’ homes	CLs	4	52y	25 % f; 75 % m	NR (1 said to be West Indian)	49y	3.5y	NR
Sharpe [28]	Unspecified, hospital	CS	1	51y	1 m	NR	50/51y	Around 11 months	‘tetrabenazine and imipramine’
Wieck et al. [27]	London, England, Neurology clinic, participants’ homes	RCT	*N* = 23 Intervention group *N* = 11; Control group N- = 12	intervention group: 52y control group: 49y	35 % f; 65 % m	NR	NR	Intervention 10y; control 13y	Psychotherapy, biofeedback and drugs.

*CS* case studies, *pRCT* pilot randomised controlled trial, *CLs* clinical study, *NR* not reported, *EMG* electromyography

^a^Average age of onset in years. ^b^Mean duration of condition, ^c^Previous medications/treatments

The studies originated from Australia [17], England [24, 26, 27] and the United States of America (USA) [21, 22]; Bell and Thompson [18], Cottraux et al. [25] and Sharpe [28] do not specify the country where their studies were conducted (Table 2).

Boyce et al. [23], Greenberg [26], Sharpe [28] and Wieck et al. [27], conducted the research in hospitals. Bell and Thompson [18] and Bell et al. [21, 22] observed putting on golf courses. Cottraux et al. [25] described their research setting as a clinical study using participants who were referred to their department. Faircloth and Reid [24] did not provide definite details of where their research was conducted (Table 2).

### Idiopathic adult onset focal dystonia

We found one study examining blepharospasm [28], two studies that investigated cervical dystonia [23, 24], three studies that conducted research with patients living with writer’s cramp [25–27] and three studies that tested an intervention for the yips [18, 21, 22] which is a type of dystonia that causes ‘jerks, tremors and spasms [and] predominately affects the distal upper extremity’ ^(p.424)^ [29]. We found the yips to occur in golfers [18, 21, 22]. Consequently, none of the identified studies included patients with generalised dystonia and therefore, the findings of this review can only report on the effectiveness of behavioural therapies for focal dystonia (Table 3).

**Table 3:** Included studies interventions

Citation and condition^a^	Intervention and control	Length of each treatment session (mins)	Frequency	Duration
Bell and Thompson [18] Golfing Yips	Intervention: SFGI protocol Control: None	20–30	2x weekly	Unclear duration noted as a number of rounds of golf with follow-up
Bell et al. [21] Golfing Yips	Intervention: SFGI protocol Control: None	20	2x weekly	Unclear duration noted as a number of rounds of golf with follow-up
Bell et al. [22] Golfing Yips	Intervention: SFGI protocol Control: None	15	2x weekly (unclear)	Unclear duration noted as a number of rounds of golf with follow-up
Boyce et al. [23] Cervical dystonia (neck dystonia)	Intervention: Exercise plus relaxation Control: Relaxation only	30 (approx.)	4x weekly for the first 4 weeks and then 8x ‘fortnightly for the following eight weeks’	8 sessions over 12 weeks
Cottraux et al. [25] Writer’s cramp (hand dystonia)	Intervention: Multimodal behaviour therapy package with or without EMG feedback Control: None	20 (EMG feedback)	2x daily (without EMG feedback), Unspecified for EMG feedback	Average total = 14.7 sessions (time period unspecified)
Faircloth and Reid [24] Cervical dystonia (neck dystonia)	Intervention: Cognitive Behavioural Therapy (CBT) approach adapting negative thoughts and beliefs into more productive thinking styles Control: None	Unspecified	Unspecified	‘9 sessions lasting 10.5 h’
Greenberg [26] Writer’s cramp (hand dystonia)	Intervention: Habit reversal with awareness training, re-education and in vivo exposure. (included encouragement from family to practice) Control: None	Unclear (for one participant 1 h is reported)	Unspecified	Average total = 4 sessions over 5.5 h
Sharpe [28] Blepharospasm (eye dystonia)	Intervention: ‘Flexible behavioural therapy approach Control: None	60	Unspecified	Approx. 17 h over 14 weeks (1 week included in-patient treatment)
Wieck et al. [27] Writer’s cramp (hand dystonia)	Intervention: Habit reversal (plus homework practice) Control: Relaxation	90	5x monthly	1 month

*EMG* electromyography, *SFGI* solution focused guided imagery

^a^ The identified studies examined different forms of the MDS idiopathic adult onset focal dystonia category

Three studies provided a full report of participants’ ethnicity [18, 21, 22]. Greenberg [26] described the ethnicity of one out of his four participants. The mean age taken across the entire sample (*N* = 73) was 47 years (range 36–57.8 years) although it is unclear whether Wieck et al. [27] included the ages of three participants who withdrew (Table 2).

Of the 73 participants 27 (37 %) were female. For five studies 100 % of the sample were male [18, 21, 22, 24, 28] (Table 2). Adherence rates for all of the identified studies were at above 50 % although sample sizes for each study were very low (see Table 4). Two RCTs looking at cervical dystonia and writer’s cramp reported slightly higher levels of adherence among the control groups (91 % for Boyce et al. [23] and 92 % for Wieck et al. [27]) compared with the intervention groups (78 % for Boyce et al. [23] and 82 % for Wieck et al. [27]). Cottraux et al. [25] described an adherence rate of 60 % among people with writer’s cramp with two participants refusing treatment and four participants withdrawing during the intervention phase. Interestingly, Cottraux et al. [25] found that three participants, who had received treatment, later withdrew from the study despite reporting handwriting improvements.

**Table 4:** summary of outcome measures and findings for each identified study

Citation and condition	Outcome measure(s) and timings	Procedure	Facilitator and evidence of facilitator training	Results & Adherence	Comments
Bell and Thompson [18] Golfing Yips	Yip frequency, baseline, during 5 rounds of golf and at a 60-day follow-up Physiological – observed yip frequency & Putting percentages.	Researcher read aloud the SGFI protocol to the participant prior to them putting.	Researcher (also, participant and playing partner recorded yip frequency and putting percentage) Training not reported	Exposure to the SFGI reduced yip frequency from an average of 9.2 yips per round to 0.2 yips per round. Only 1 yip occurrence was recorded during the intervention phase. At baseline, the participant averaged 77 % putts within four feet compared with 97 % during the intervention phase. The participant averaged 81 % of putts within four feet or less at the 60-day follow-up. While the participant said that they felt more confident to putt after being exposed to SFGI, they were unsure why it had helped and were worried that their yips would reoccur. 100 % adherence	There was no control group to compare the findings, although the authors note that ‘each participant serves as their own control as participants’ performance is compared across baseline and intervention phases’ The study recruited only one participant and so no statistical power, generalisability or reliability. Results open to reporter bias. Yip type was unspecified.
Bell et al. [21] Golfing Yips	Yip occurrences were observed at baseline, during at least 5 treatment sessions of golf and at a 3-week follow-up golfing session after the last SFGI round. Physiological – yip occurrences and percentage of putting with the yips.	1-2 independent observers were stationed at each site. The primary researcher read aloud the SFGI protocol to participants prior to them putting.	Primary researcher (also, 3 trained observers recorded putting behaviour and yip frequency)	Participant 1, BL yips 4, Reduced to 1.4, FU NR Effect size, moderate (0.65). Participant 2, BL yips 3, Reduced to 1.3 FU NR Effect size, moderate (0.55). Participant 3, BL yips 3.6, Reduced to 0.8 FU NR Effect size, moderate (0.73). (1 participant withdrew after completing four rounds of golf at baseline)	It is unclear whether the primary researcher was 1 of the 3 trained observers. Training provision was specified for the 3 observers but not for the primary researcher. The study is underpowered because it reports on only 3 participants. No control group to compare. No reported p values. The author’s note that no attempt was made to exclude Type I or Type II yips and so some or all of the participants may have been living with secondary yip dystonia.
Bell et al. [22] Golfing Yips	Yip frequency was measured at baseline, immediately after treatment and at follow-up (12–14 weeks post-treatment). Physiological – symptom severity, looking at the frequency of yip occurrences. Observations of the golfers putting were video-recorded.	Individual/1 facilitator read aloud the SGFI protocol to each participant and then recorded the participant’s answers 15 min prior to them putting.	Trained facilitators (individual/1 facilitator per participant).	100 % of participants showed a decrease in the frequency of yip behaviour: At baseline (average Mean of yip frequency): 2.86 Treatment stage (average Mean of yip frequency): 0.68 Maintenance - at least 12 weeks from treatment stage (average Mean of yip frequency): 0.08. Effect sizes reported as either ‘large’ or ‘medium’ for baseline and treatment and baseline and maintenance. 100 % Adherence	The study is underpowered because it reports on only 4 participants. No control group to compare. No reported p values.
Boyce et al. [23] Cervical dystonia (neck dystonia)	Baseline (week 0), during treatment (week 6), post-treatment (week 12) and at a 4-week follow-up (week 16). Toronto Western Spasmodic Torticollis Rating Scale (TWSTRS) Participants video-recorded and 2 neurologists masked to treatment allocation evaluated symptom severity (primary outcome). Self-rated pain and disability. Toronto Western Spasmodic Torticollis Rating Scale (TWSTRS) (secondary outcomes). depression and quality of life The Beck Depression Inventory Craniocervical Dystonia Questionnaire 24 (secondary outcomes). Cervical range of motion was recorded using a Cervical Range of Motion device (secondary outcome). Attendance at the physiotherapy sessions, adverse effects and muscle soreness were also documented. Participants completed diaries	Intervention: ‘Active neck exercises plus [whole body] relaxation’ Control: Whole body relaxation programme	Physiotherapist (individual/supervised physiotherapy sessions) Participants were also encouraged to practise exercises at home No mention of training	No adverse effects reported. Mild muscle soreness was reported in 66 % of the sample. No significant differences between intervention and control group at treatment and follow-up phases. The intervention group showed an (non-significant) improvement in depression scores compared to the control group in week 12 and ‘a greater (non-significant) improvement in TWSTRS’ in weeks 12 and 16 (p. 6). Effect size: −1.9 (reported difference between the intervention and control groups on TWSTRS). The physiotherapy and home exercise sessions were ‘well-adhered to by both groups’ Adherence 78 % intervention 91 % control	Small sample and effect size. Confounding variable: N = 7 received Botulinum Toxin therapy injections which may have reduced the effect size. No results were found to be significant. The authors note that the Cervical Range of Motion instrument has unknown reliability for people with cervical dystonia and head tremor/jerks.
Cottraux et al. [25] Writer’s cramp (hand dystonia)	Writing quality was observed at baseline (pre)-intervention), post-intervention (last session) and follow-up (at varying weeks between 1 to 9 months). Participants copied a standardised piece of text in the clinic and recorded the amount of cramps they experienced. An independent evaluator also rated participants’ frequency of spasms, writing improvement and writing quality using 3 separate scales across baseline, treatment and follow up phases.	‘Relaxation, and/or systematic desensitisation (SD), and/or assertiveness training through role playing were combined with Electromyography (EMG) feedback’ (2 participants were not given EMG feedback) (P. 182)	Unspecified but participants were referred to authors’ department No mention of training	69 % of the sample (*N* = 9) showed improvements in writing at follow-up (time at follow-up varied between participants, ranging from 1 to 9 months) Of the 4 participants who withdrew during the treatment phase, 3 showed an improvement in handwriting. Adherence 60 % (2 participants refused treatment and 4 withdrew during the treatment phase)	Small sample size. Limited results and follow-up data to draw meaningful conclusions. 7 participants were reported as presenting with severe mental health problems prior to data collection. 2 participants felt depressed or worried despite showing an improvement on the handwriting scale. 1 participant was described as having ‘’normal handwriting’ out of the 69 % who reported an improvement
Faircloth and Reid [24] Cervical dystonia (neck dystonia)	Measures were conducted at baseline (pre-treatment), post-treatment and at one, three and six month follow-ups. The participant was asked to record, using visual analogue scales, how many times they spent worrying about the physical and emotional aspects of their dystonia. The Beck Depression Inventory and the Beck Anxiety Inventory measured the participant’s overall psychological health.	Enabling self-focus, generating adaptive beliefs and challenging negative thinking.	Unspecified	Improvements were noted in psychological well-being (i.e., anxiety and depression). Pain and discomfort in the participant’s neck was reported as less severe. All of the participant’s 6 months follow-up scores were lower than at baseline.	Small study (*N* = 1) and so not statistically generalisable. Only subjective (validated and unvalidated) self-report measures were used.
Greenberg [26] Writer’s cramp (hand dystonia)	Measures were conducted at baseline (pre-treatment), 1 month, 6 months and various time periods between 2 and 6 years. Patient and therapist independently rated how severely the patient’s problem affected their everyday life and how it impacted on their ability to achieve their targets. Patients were observed during the clinic to assess their writing rate. The patient measured their writing abilities outside of the clinical hospital and where possible a friend or relative confirmed the findings. Fear and anxiety were also rated using general, validated measures.	A combination of ‘habit reversal, in vivo exposure and re-education’ [apart from 1 participant] Awareness training, encouragement from relatives and homework practice were also given.	Nurse therapists, family and friends supervised homework practice and offered encouragement the nurse therapists received 1–2 sessions of teaching in habit reversal therapy	The treatment showed an improvement in writing skills at least until the 6-months follow-up phase (p. 297). All 4 participants responded to the treatment. 1 participant relapsed after stopping habit reversal practice. 100 % Adherence	Small sample size. Not all of the measures were observed. An independent assessor did not assess tidiness. Therapists only observed handwriting in artificial settings. ‘The habit reversal adopted in this trial – extension of the wrist, fingers, and thumb – inevitably means that the pen is released for 5 s. The spasm could thus be said to be rewarded by avoidance’ 1 participant was reported as experiencing high levels of anxiety.
Sharpe [28] Blepharospasm (eye dystonia)	Unspecified, baseline, treatment and 9 months follow-up	Relaxation (exercise) training for the eyelids, learning not to force the eyelids open and seeking rewarding reinforcements for keeping the eyelids open	Unspecified	Outcomes included reduction of eyelid spasm and ache as well as relaxation of the eyelids. Participant was able to control spasms and resume daily social and business activities. No deterioration was reported at the 9-months follow-up. 100 % Adherence	Small study (*N* = 1) and so not statistically generalisable. Minimal description of follow-up procedures or outcome measures.
Wieck et al. [27] Writer’s cramp (hand dystonia)	Outcome measures were recorded at baseline (pre-treatment, weeks 0 and 4), post-treatment (week 8) and at a 3 months follow-up (week 20). ‘Observation of writing within the session, assessment of writing tasks completed at home, and blind ratings by an independent assessor’	Patients were educated to know when their symptoms were triggered and to immediately respond (e.g., by putting down the pen) until their cramp stopped. Severer participants were asked to draw lines proceeding to more complex shapes and finally, words. Control group trained to practice ‘progressive relaxation technique’ (p. 113).	2 therapists No mention of training	No significant differences were reported between the intervention and control groups on 89 % of the measures. There was an increase in the frequency of words written at home among the relaxation group than with the habit reversal group (where there was a slight decrease). Both groups improved significantly ‘in legibility, number of interruptions, and the ratings for problem severity, pain and difficultly’ (p. 114). However, this was only slightly and participants ‘remained substantially handicapped’ with regards to writing speed (p. 114). The authors conclude that they found no evidence to suggest that habit reversal treatment is more effective than general relaxation. Adherence 82 % intervention 92 % control	Small sample size. *N* = 1 experienced anxiety and avoidance behaviour and *N* = 3 experienced generalised anxiety

Three studies looking at writer’s cramp reported participants were living with various emotional disturbances [25–27]. Of these three studies, two [25, 27] examined patients’ physical and mental health. However, Cottraux et al. [25] only screened 10 patients (out of the 15 originally recruited into the study) using Multiple Personality Inventory (MMPI) referred to as MimiMult. While participants who were screened with MMPI were not reported as presenting with a consistent pathological pattern, Cottraux et al. [25] noted that seven individuals reported chronic emotional or sexual disturbances. Furthermore, all participants had previously used various treatments including tranquillizers, bromocriptine and psychotherapy [25]. Greenberg [26] reported that one participant out of four displayed general anxiety symptoms. Wieck et al. [27] described one participant in their study as anxious and displaying avoidance behaviour and two patients were reported with living with generalised anxiety.

Bell et al. [21, 22] did not specify how long their participants had been living with dystonia. Sharpe [28] was the only author who reported that the participant had been living with dystonia for under a year (10 months), stating that the participant had also experienced dystonia symptoms for four weeks 11 months earlier. The average duration of dystonia in the remaining six studies was seven years.

### Interventions

All of the identified studies examined various behavioural therapies. These include SFGI protocol to individuals experiencing yips [18, 21, 22], which typically causes spasms in the upper limb(s) and can occur in various sports including golf [29], combined behavioural and physical practice including relaxation for patients with cervical dystonia [23], SD, assertiveness training and/or EMG feedback on patients with writer’s cramp [25] and CBT for cervical dystonia [24]. Greenberg [26] investigated the effectiveness of habit reversal therapy (which aims to reduce spasm and involuntary movements by delivering fine motor techniques that are opposite to the involuntary contraction) with awareness training, re-education, and ‘in vivo exposure in which a hierarchy of situations was constructed in which cramp was increasingly likely to occur.’^(p.234)^ Sharpe [28] examined the effects of relaxation with conditioning therapy for one patient with blepharospasm. Finally, Wieck et al. [27] investigated writer’s cramp and assigned the control group individually tailored sessions of relaxation and the intervention group, habit reversal therapy. While the studies’ interventions were fairly diverse, they all focused on examining the effectiveness of enabling participants to self-manage their disability outside of the clinical setting [18, 21–28].

### Outcome measures

Eight studies reported outcome measures utilising independently assessed and/or facilitator-led ratings and/or self-report un-validated and/or validated measures to evaluate physiological and/or psychological improvement [18, 21–27].

Two studies used the Beck Depression Inventory to assess depression scores [23, 24]. Faircloth and Reid [24] also used un-validated self-report measures to evaluate psychological wellbeing and an un-validated measure to investigate physical functioning. The authors did not objectively measure any clinical outcomes [24].

The studies examining writer’s cramp observed writing frequency among participants in and outside of the clinic [25–27]. However, Greenberg [26] was the only author examining writer’s cramp to not use an independent assessor to verify handwriting improvements. Instead, only patients and therapists involved in treatment delivery rated handwriting quality [26]. Cottraux et al. [25] utilised an independent evaluator to rate spasm frequency, handwriting quality and handwriting improvement on three separate scales that were combined to obtain the overall mean value of each participant’s handwriting improvement score. Since these data were only obtained in the clinic participants were also asked to self-evaluate their handwriting in a non-clinical setting [25]. However, data obtained from participants outside of the clinic were not systematically scored [25].

Having applied the SFGI protocol, Bell and Thompson [18] requested that the participant and a playing partner record yip behaviour during putting. Similarly, Bell et al. [21] used trained observers to watch putting behaviour among the participants. Video observations of putting behaviour were used by Bell et al. [22] to increase inter-reliability. Bell and Thompson [18] and Bell et al. [21, 22] also used the SFGI protocol to ask participants questions about the severity of their dystonia.

Sharpe [28] did not report using any outcome measures. The authors of this review were unable to contact Sharpe [28] given how long ago the study was published. Nonetheless, Sharpe [28] provides details about outcomes post-treatment and at the 9-month follow-up.

## Discussion

We found very limited evidence for behavioural interventions in the treatment and management of idiopathic adult onset dystonia. All of the studies we identified were for the MDS idiopathic adult onset focal dystonias group. The findings therefore only apply to this group. There were some promising results from the RCTs and uncontrolled studies that behavioural therapies can improve peoples’ physical and emotional well-being. Some of the underlying mechanisms mapped onto the behavioural interventions specifically included action planning and problem solving to improve productivity and overall management, education and behavioural experiments to empower and help control symptoms and education and distraction to manage anxiety through cognitive restricting to lessen anxiety and also the severity of dystonia. However all of the identified studies used small samples and nearly all had substantial methodological weaknesses which precluded a rigorous meta-analysis. Therefore, none of the findings can be generalised beyond the study setting. The available evidence applies only to focal dystonias and we cannot make any comment based on possible effectiveness from empirical data for dystonias affecting more than one body segment. Nevertheless, the theoretical justification for behavioural interventions is equally applicable to more than just focal dystonias. This means that the very limited empirical data might be applicable to a wider population of those affected by dystonia. A possible reason for why there have been very few research studies examining the effectiveness of behavioural therapies for people with dystonia could be because idiopathic adult onset dystonia is often misdiagnosed as an emotional illness limiting acceptance of behavioural interventions [9].

There have however been other studies which have also shown that CBT could be beneficial with other movement disorders. For example in a pilot study, tailored CBT for this with depression and Parkinson disease showed a reduction in negative cognitions, reduced depressive symptoms and an increase in the sense of social support [30].

### Strengths and limitations of the review

The authors of this review employed a rigorous search and screening strategy to identify and evaluate the relevant studies. It has highlighted an important gap in the literature and is one of the first reviews too examine behavioural interventions for the management of dystonia.

Since Sharpe’s [28] study was conducted some time ago the review authors were unable to obtain information about what outcome measures he utilised. Furthermore, the review found no studies examining the effects of behavioural therapies in people with generalised dystonia. Consequently, this review is limited in not being able to comment whether behavioural treatments would be effective for this patient group. Conducting a meta-analysis was not possible because of study heterogeneity.

### Future research and practice

Future research is necessary to establish the effects, if any, of behavioural interventions in patients living with idiopathic adult onset dystonia. Consequently, researchers should consider RCTs with powered sample size, clearly specifying the diagnostic process involved and using physiological and psychological validated outcome measures to capture changes. Detailing the severity of patients’ dystonia could also be useful for comparing treatment effectiveness across different levels of dystonia.

Future research should also examine generalised dystonia in relation to behavioural therapies to address the current gap in the medical and psychology literature.

It is only by addressing the many challenges in designing robust studies of behavioural interventions for the dystonias that we can find out if behavioural treatments can help these stigmatising and disabling chronic disorders.

## Conclusion

In conclusion, this review identified nine studies all with low methodological quality, making it difficult to establish the effectiveness of behavioural interventions in patients living with idiopathic adult onset dystonia. The limited data indicates, however, that behavioural therapies could have a beneficial effect for enabling individuals to manage their dystonia, particularly when combined with relaxation practice [23, 25, 27, 28]. Further high-quality research is needed to fully assess the effectiveness of holistic behavioural therapies in patients with idiopathic adult onset dystonia.

## Appendix

**Table 5:** Criteria for quality assessment

	Risk of bias rating			
	Low	Moderate	High	Unclear
Diagnostic process	Diagnosis according to established, published criteria	Diagnostic process is described in sufficient detail, but no published criteria were used	Authors mention who performed the diagnosis (e.g., experienced movement specialists) but do not describe the diagnostic process itself	Diagnostic process not described
Sample size	≥ 100 per group (sufficient to detect an effect of Cohen‘s d=0.35)	21-99 per group	< 20 per group (not sufficient to detect a sample size of Cohen‘s d = 0.8)	Sample size not mentioned
Control group	Healthy controls matched for age and sex	Unmatched healthy controls, matched only for age or only for sex, historical control group, controls with another disorder but no healthy controls	No control group	Not mentioned whether control group was included
Physiological/ psychological measure	Valid physiological and/or psychological measures of subjective *and* objective measures (for example clinician ratings of dystonia severity according to validated scale and/or psychological wellbeing *and* one validated self-report questionnaire)	One adequate physiological and/or psychological measure (for example clinician ratings of dystonia severity according to validated scale and/or psychological wellbeing *or* one validated self-report questionnaire)	Unvalidated subjective and/or objective measures, questionnaire insensitive or otherwise inappropriate	Measures not sufficiently described
Description of intervention	An adequate, clear and comprehensive description of the intervention is provided, including the following: what is being tested, theoretical framework underlying the intervention, components of the intervention, how it was delivered and by whom, what research methods were employed, analytical techniques, when and where it was delivered, whether any materials were used and a description of any relevant facilitator/researcher training	Partially full descriptions of the intervention are provided (i.e. between 1-5 components of this criterion lack sufficient detail or are not provided but the remaining 6-11 components are described in detail)	Very few details are given about how the intervention was delivered (e.g. between 1-5 components of this criterion are adequately described but the remaining 6-11 are not described in detail and/or missing) or otherwise inappropriate or irrelevant descriptions are provided	None of the criterion’s components are described
Medication	Un-medicated sample	Medicated sample, medication reported in detail	Medicated sample, medication insufficiently described	Medication not described
Statistics	Analysis/statistical approach is adequate for design and sample size, conditions for use of statistical approach tested and described in sufficient detail	Minor shortcomings leading to imprecision, but not invalidation of the results, e.g. conditions for use of statistical approach not tested or not described	Major shortcomings, e.g. no significance testing, inappropriate statistical procedure	Statistical approach not sufficiently described

Adapted from Hertenstein et al. [18]

## Abbreviations

CBT: cognitive behavioural therapy
EMG: electromyography
MDS: Movement Disorder Society
MMPI: multiple personality inventory
RCTs: randomised controlled trials
SD: systematic desensitisation
SFGI: solution-focused guided imagery
TENS: transcutaneous electrical nerve stimulation
TWSTRS: Toronto Western Spasmodic Torticollis Rating Scale
USA: United States of America
